# Bark Extracts of *Chamaecyparis obtusa* (Siebold & Zucc.) Endl. Attenuate LPS-Induced Inflammatory Responses in RAW264.7 Macrophages

**DOI:** 10.3390/plants14152346

**Published:** 2025-07-29

**Authors:** Bo-Ae Kim, Ji-A Byeon, Young-Ah Jang, Yong-Jin Kwon

**Affiliations:** 1 Department of Cosmetics, College of Health and Safety Science, Mokwon University, Daejeon 35349, Republic of Korea; kba@mokwon.ac.kr; 2 Department of Cosmeceutical Science, Kyungsung University, Busan 48434, Republic of Korea; bzia@ks.ac.kr; 3 Department of Cosmetic Science, Daegu Haany University, Gyeongbuk 38540, Republic of Korea; yajcosmetic@dhu.ac.kr; 4 Department of Cosmetic Science, Kyungsung University, Busan 48434, Republic of Korea

**Keywords:** *Chamaecyparis obtusa* (Siebold & Zucc.) Endl. bark extract, anti-inflammatory response, inducible nitric oxide synthase (iNOS), cyclooxygenase-2 (COX-2), nitric oxide (NO), prostaglandin E_2_ (PGE_2_)

## Abstract

*Chamaecyparis obtusa* (Siebold & Zucc.) Endl. (*C. obtusa*) is an evergreen conifer native to temperate regions such as South Korea and Japan, traditionally used for its anti-inflammatory properties. However, the molecular mechanisms underlying the anti-inflammatory effects of *C. obtusa* bark extracts remain poorly understood. In this study, I compared the biological activities of *C. obtusa* bark extracts prepared using boiling water (COWB) and 70% ethanol (COEB), and investigated their anti-inflammatory mechanisms in lipopolysaccharide (LPS)-stimulated RAW264.7 macrophages. COEB significantly suppressed both mRNA and protein expression of inducible nitric oxide synthase (iNOS) and cyclooxygenase-2 (COX-2), along with decreased production of their respective inflammatory mediators, nitric oxide (NO) and prostaglandin E_2_ (PGE_2_). Additionally, COEB selectively downregulated interleukin (IL)-1β expression, without affecting tumor necrosis factor-α (TNF-α), and unexpectedly upregulated IL-6. Notably, COEB did not inhibit the LPS-induced activation of major inflammatory signaling pathways, including mitogen-activated protein kinase (MAPK), nuclear factor-kappa B (NF-κB), and Janus kinase/signal transducer and activator of transcription (JAK/STAT). These findings suggest that COEB exerts anti-inflammatory effects by modulating key inflammatory mediators independently of canonical signaling pathways and may offer a novel therapeutic strategy for controlling inflammation.

## 1. Introduction

Inflammatory responses are fundamental defense mechanisms by which the body reacts to infection or tissue injury, involving the recruitment of immune cells to the affected site to eliminate pathogens and initiate tissue repair [1]. However, when inflammation becomes chronic or excessive, it can contribute to the development of various immune-related disorders, including atopic dermatitis, psoriasis, asthma, and autoimmune diseases [2,3,4]. Therefore, elucidating the molecular mechanisms underlying inflammation is critical for developing effective strategies to regulate inflammatory responses.

Toll-like receptor 4 (TLR4) plays a pivotal role in the innate immune response by acting as a primary sensor for pathogen-associated molecular patterns (PAMPs), such as lipopolysaccharide (LPS) derived from Gram-negative bacteria [5,6]. Upon LPS recognition, TLR4 triggers a signaling cascade that activates macrophages, the first line of defense in the immune system [5]. This activation leads to the upregulation of various pro-inflammatory genes, including inducible nitric oxide synthase (iNOS) and cyclooxygenase-2 (COX-2), resulting in the production of key inflammatory mediators such as nitric oxide (NO) and prostaglandin E_2_ (PGE_2_) [7]. These secretory molecules contribute to the amplification of inflammatory responses and play an essential role in host defense [8]. Several intracellular signaling pathways regulate these processes, among which the mitogen-activated protein kinase (MAPK), nuclear factor kappa B (NF-κB), and Janus kinase/signal transducer and activator of transcription (JAK/STAT) pathways are critically involved [9]. These pathways mediate the transcriptional activation of numerous inflammatory cytokines and enzymes, thereby orchestrating the overall inflammatory response.

Anti-inflammatory agents suppress the production of inflammatory mediators by inhibiting inflammation-related gene expression or blocking the signaling pathways that regulate them [10,11]. Proper modulation of these mechanisms helps alleviate inflammation, minimize tissue damage, and ultimately improve symptoms of various inflammatory diseases [12]. Notably, anti-inflammatory compounds derived from natural products may act through distinct mechanisms compared with conventional synthetic drugs, offering promising potential as novel therapeutic candidates [13,14,15]. Therefore, elucidating the anti-inflammatory mechanisms of natural products is essential for the development of new preventive and therapeutic strategies against inflammatory diseases.

*Chamaecyparis obtusa* (Siebold & Zucc.) Endl. (*C. obtusa*, Cypress family) is an evergreen conifer widely cultivated in temperate regions such as South Korea and Japan [16]. Traditionally, various parts of this plant—including its leaves, wood, and bark—have been used in folk medicine for their therapeutic effects, including anti-inflammatory, wound healing, anti-cancer, and anti-melanogenic properties [17,18,19,20,21,22]. The bark of *C. obtusa* was selected as the extraction material in this study because of its distinctive phytochemical composition and potential for bioactive applications. As the plant’s outermost protective tissue, the bark is known to accumulate high levels of phenolic compounds, including polyphenols, flavonoids, and lignin, which are strongly associated with antioxidant and anti-inflammatory effects [23]. In contrast, previous studies have shown that the leaves and stems of *C. obtusa* primarily contain volatile terpenoids and essential oils such as α-terpineol and sabinene, with relatively limited focus on phenolic compounds relevant to inflammation [24,25]. Moreover, bark is often discarded as a by-product in the lumber industry, making it a sustainable and cost-effective resource for developing functional materials. These characteristics support the rationale for selecting bark as a promising source of anti-inflammatory agents.

In this study, both boiling water and 70% ethanol were used as extraction solvents to effectively isolate and compare the bioactive compounds present in *C. obtusa* bark. Boiling water extraction is suitable for obtaining highly polar, water-soluble components such as polysaccharides and certain phenolic acids, and has been widely used in the food and pharmaceutical industries [26,27,28]. In contrast, 70% ethanol is a hydroalcoholic solvent with intermediate polarity, known to efficiently extract a wide range of bioactive compounds, including flavonoids and polyphenols, and is commonly used as a standardized method for natural product extraction [29]. Furthermore, previous studies have reported that most of the active compounds in *C. obtusa* bark are phenolic in nature and are more abundantly extracted with ethanol or ethyl acetate than with water [23]. Thus, this dual-solvent extraction approach is scientifically valid for evaluating the differences in phytochemical composition and biological activity between the two extracts.

This study aimed to investigate the anti-inflammatory effects of bark extracts from *C. obtusa* by comparing the biological activities of extracts obtained using 70% ethanol and boiling water. In addition, I sought to clarify their underlying mechanisms of action and evaluate their potential as natural anti-inflammatory therapeutic agents.

## 2. Results

### 2.1. Fingerprint Analysis of C. obtusa Bark Extracts

**Figure 1:**
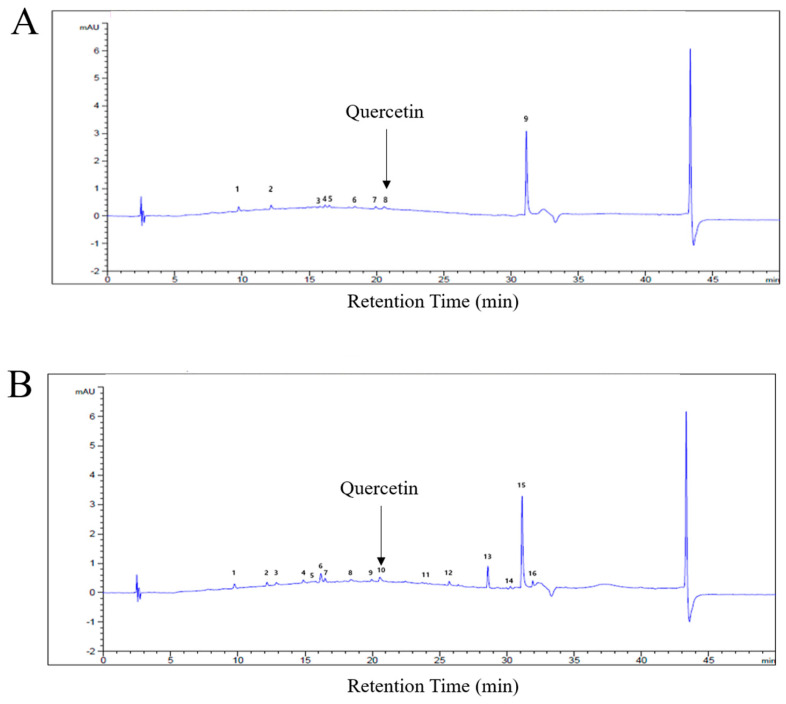
Fingerprint analysis of *C. obtuse* bark extracts. (**A**,**B**) HPLC chromatograms showing the chemical fingerprint profiles of COWB (**A**) and COEB (**B**).

To characterize the chemical composition of *C. obtusa* bark extracts, fingerprint analysis was performed using high-performance liquid chromatography (HPLC). Chromatograms of both COWB and COEB were obtained, showing distinct chemical profiles (Figure 1A,B). Based on previous findings that tree bark, including that of *C. obtusa*, is rich in flavonoids such as quercetin [23], quercetin was selected as a standard marker compound for HPLC analysis (Supplementary Figure S1). Quantitative analysis revealed the presence of quercetin in both extracts, with a slightly higher content detected in COEB. This observation aligns with earlier reports that 70% ethanol is an effective solvent for extracting polyphenols and flavonoids from plant materials because of its intermediate polarity [29].

### 2.2. Cytotoxicity and Anti-Inflammatory Effects of COWB and COEB on LPS-Stimulated RAW264.7 Cells

**Figure 2:**
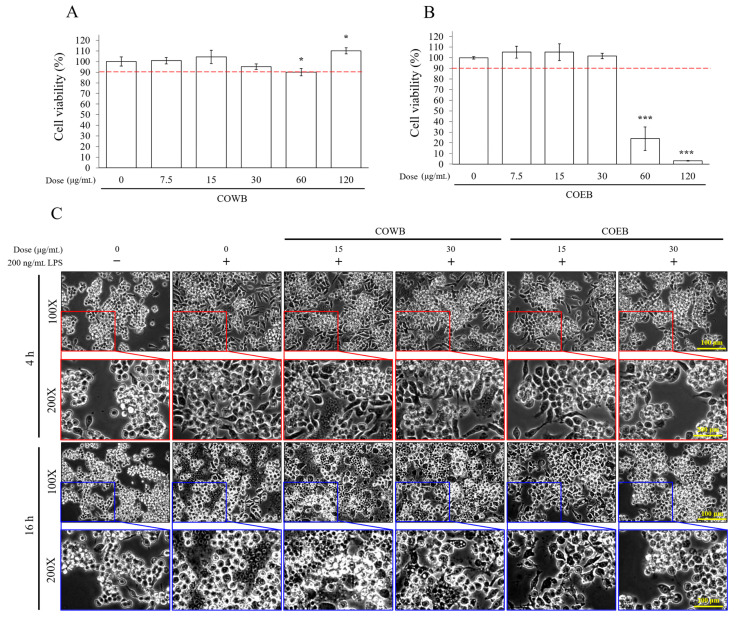
Cell cytotoxicity and morphologic change by COWB and COEB in RAW264.7 cells. (**A**,**B**) Cells were seeded into 96-well plates and treated with COWB (**A**) or COEB (**B**) for 24 h. Cell viability was assessed using the MTT assay. The red dotted line indicates 90% cell viability. (**C**) Cells were seeded into 6-well plates, pre-treated with COWB or COEB for 2 h, and then stimulated with LPS for 16 h. All data are presented as mean ± SD (*n* = 3). Statistical significance was determined using an unpaired Student’s *t*-test. *p* < 0.05 = *, *p* < 0.001 = ***.

Before conducting the anti-inflammatory efficacy experiments, the cytotoxicity of *C. obtusa* bark extracts was assessed in RAW264.7 macrophages. COWB showed no significant cytotoxicity up to 120 μg/mL, indicating high cellular safety (Figure 2A). In contrast, COEB was non-toxic up to 30 μg/mL but induced significant cytotoxicity at 60 and 120 μg/mL (Figure 2B). Based on these results, non-cytotoxic concentrations of 15 and 30 μg/mL were selected for subsequent anti-inflammatory experiments. LPS stimulation is known to induce characteristic morphological changes in macrophages, including initial elongation and subsequent swelling over time [30]. These morphological alterations serve as visual markers of inflammation. Upon LPS treatment for 4 or 16 h, RAW264.7 cells exhibited pronounced elongation and swelling. COWB did not noticeably prevent these LPS-induced changes, whereas COEB effectively reduced them (Figure 2C). These findings suggest that COEB more effectively attenuates LPS-induced morphological alterations than COWB at non-cytotoxic concentrations, suggesting its superior anti-inflammatory potential.

### 2.3. Comparison of Anti-Inflammatory Effects of COWB and COEB in LPS-Stimulated RAW264.7 Cells

**Figure 3:**
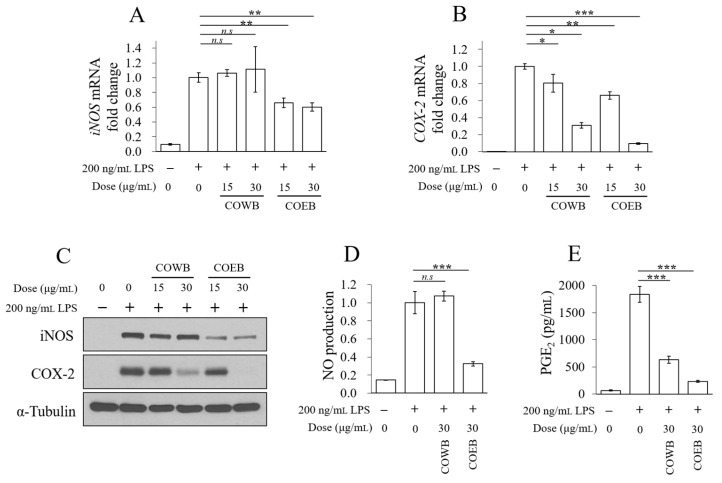
Comparison of anti-inflammatory effects of COWB and COEB in LPS-stimulated RAW264.7 cells. (**A**–**E**) Cells were seeded in 6-well plates, pre-treated with COWB or COEB for 2 h, and then stimulated with LPS for 16 h. (**A**,**B**) mRNA expression levels of iNOS (**A**) and COX-2 (**B**) were evaluated by qPCR. C. Protein expression levels of iNOS and COX-2 were analyzed by Western blotting. (**D**,**E**) Production of NO (**D**) and PGE_2_ (**E**) was measured using the Griess reagent and a PGE_2_ ELISA kit, respectively. All data are presented as mean ± SD (*n* = 3). Statistical significance was determined using an unpaired Student’s *t*-test. *p* < 0.05 = *, *p* < 0.01 = **, and *p* < 0.001 = ***. *n.s.* = not significant.

The protein and mRNA expression levels of iNOS and COX-2, key enzymes involved in the inflammatory response, were analyzed following treatment with COWB and COEB. The mRNA expression of iNOS was not reduced by COWB, whereas COEB significantly suppressed its expression in LPS-stimulated RAW264.7 cells (Figure 3A). For COX-2, both COWB and COEB significantly inhibited mRNA expression, with COEB showing a more pronounced effect (Figure 3B). Consistent with the mRNA results, COEB more effectively downregulated LPS-induced iNOS and COX-2 protein expression compared with COWB (Figure 3C). Furthermore, COEB more effectively reduced the production of NO and PGE_2_ than COWB (Figure 3D,E). These findings suggest that COEB exhibits superior anti-inflammatory activity by more effectively inhibiting iNOS and COX-2 expression and reducing their downstream mediators.

### 2.4. COEB Regulates LPS-Induced Pro-Inflammatory Cytokines

**Figure 4:**
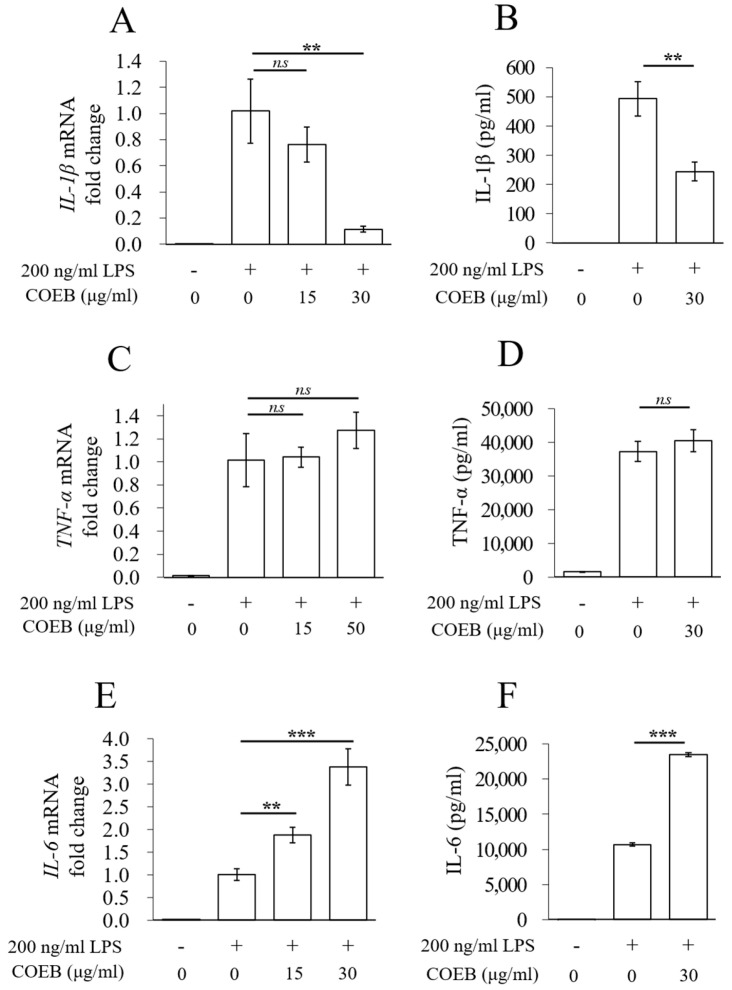
COEB regulates LPS-induced pro-inflammatory cytokines. (**A**–**F**) Cells were seeded in 6-well plates, pre-treated with COWB or COEB for 2 h, and then stimulated with LPS for 4 or 8 h. (**A**,**B**) mRNA expression levels of IL-1β were analyzed by qPCR (**A**), and protein levels of IL-1β were measured using an ELISA kit (**B**). (**C**,**D**) mRNA expression levels of TNF-α were analyzed by qPCR (**C**), and protein levels of TNF-α were measured using an ELISA kit (**D**). (**E**,**F**) mRNA expression levels of IL-6 were analyzed by qPCR (**E**), and protein levels of IL-6 were measured using an ELISA kit (**F**). All data are presented as mean ± SD (*n* = 3). Statistical significance was determined using an unpaired Student’s *t*-test. *p* < 0.01 = **, and *p* < 0.001 = ***. *n.s.* = not significant.

Excessive secretion of pro-inflammatory cytokines in macrophages is a key factor contributing to inflammatory diseases [31]. To further investigate the anti-inflammatory effects of COEB, the mRNA expression and secretion levels of IL-1β, TNF-α, and IL-6 were analyzed. COEB significantly reduced IL-1β mRNA expression and secretion, expecting suppression of early inflammatory responses (Figure 4A,B). However, TNF-α mRNA expression and secretion remained unchanged by COEB (Figure 4C,D). Notably, COEB increased IL-6 mRNA expression and secretion compared with the LPS-only group (Figure 4E,F). These findings suggest that while COEB effectively attenuates inflammation by suppressing iNOS, COX-2, and IL-1β, it may exert differential effects on specific cytokine profiles, particularly IL-6.

### 2.5. The Anti-Inflammatory Effects of COEB Are Independent of NF-κB, MAPK, and JAK/STAT Signaling Pathways

**Figure 5:**
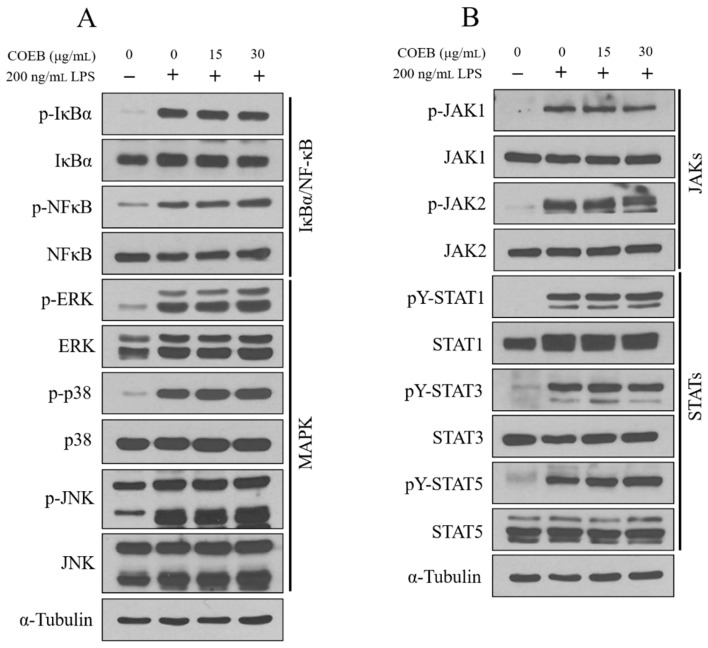
The anti-inflammatory effects of COEB are independent of NF-κB, MAPK, and JAK/STAT signaling pathways. (**A**,**B**) Cells were seeded in 6-well plates and pre-treated with COWB and COEB for 2 h, then treated with LPS for 4 h. Activation of Iκ-Bα/NF-κB ((**A**), top panel), MAPK ((**A**), bottom panel), and JAK/STAT (**B**) signaling pathways was analyzed by Western blotting.

LPS stimulation activates key inflammatory signaling pathways in macrophages, including MAPK, NF-κB, and JAK/STAT, all of which are known to regulate various inflammatory responses [32]. To further elucidate the anti-inflammatory mechanism of COEB, its effects on these upstream signaling pathways were investigated. The results showed that COEB did not inhibit the LPS-induced activation of NF-κB or IκB-α (Figure 5A top panel). Similarly, COEB had no observable effect on the phosphorylation of MAPK family members, including p38, JNK, and ERK (Figure 5A bottom panel). Moreover, the phosphorylation levels of JAK1, JAK2, STAT1, STAT3, and STAT5 remained unaffected by COEB (Figure 5B). These findings indicate that the anti-inflammatory activity of COEB occurs independently of the MAPK, NF-κB, and JAK/STAT signaling pathways.

## 3. Discussion

The inhibition of iNOS and COX-2 expression is considered a critical marker for evaluating anti-inflammatory efficacy, as these enzymes are responsible for producing NO and PGE2, which are key mediators in the amplification of inflammatory responses [7]. In our results, COEB showed a dose-dependent suppression of both iNOS and COX-2 at the mRNA and protein levels, which was consistent with the reduction in NO and PGE2 concentrations. This indicates that COEB exerts its anti-inflammatory effects not only at the transcriptional level but also through downstream regulation of inflammatory mediators.

In addition to these classical markers, I evaluated the regulation of pro-inflammatory cytokines [31]. Interestingly, COEB significantly reduced the expression and secretion of IL-1β but did not affect TNF-α levels. Moreover, COEB unexpectedly increased IL-6 expression and secretion. Although IL-6 is generally regarded as a pro-inflammatory cytokine, its role is multifaceted. Under certain physiological conditions, IL-6 contributes to tissue repair, regeneration, and immune homeostasis [33,34]. Therefore, the increase in IL-6 observed following COEB treatment may not solely represent a detrimental inflammatory response but could instead indicate a broader immunoregulatory effect. This finding highlights the need for further investigation using COEB to clarify the biological significance and functional consequences of IL-6 upregulation.

To further explore the mechanistic basis of COEB’s effects, I examined key intracellular signaling cascades known to mediate inflammatory responses, namely the NF-κB, MAPK (p38, ERK, JNK), and JAK/STAT pathways [9]. Surprisingly, COEB did not inhibit the phosphorylation of these canonical signaling proteins, indicating that its anti-inflammatory activity may occur independently of these inflammatory pathways. This finding contrasts with the mechanism of many synthetic and natural anti-inflammatory agents, which typically exert their effects by directly targeting these canonical pathways. Therefore, COEB may exert its anti-inflammatory activity through non-canonical mechanisms, such as the PI3K/Akt, Nrf2/HO-1, or TGF-β pathways, or potentially via epigenetic regulation [35,36,37,38]. Particularly, the Nrf2/HO-1 pathway has been demonstrated to play a central role in regulating inflammatory responses. For instance, Dihydroquercetin significantly attenuated LPS-induced production of pro-inflammatory cytokines in RAW264.7 macrophages by activating the AMPK/Nrf2/HO-1 axis, and the anti-inflammatory effects were abolished when HO-1 expression was silenced via siRNA [39]. Similarly, Nardochinoid C alleviated LPS-mediated inflammation by promoting nuclear translocation of Nrf2 and inducing HO-1 expression, while pharmacological inhibition of HO-1 completely abrogated its protective effects [40]. Further investigation is warranted to clarify these alternative pathways.

These findings raise the possibility that COEB modulates inflammatory mediators without disrupting upstream signaling pathways, potentially allowing for the maintenance of essential immune functions while reducing excessive inflammatory responses. The selective cytokine modulation by COEB, particularly the suppression of IL-1β alongside the preservation or enhancement of IL-6, may offer therapeutic advantages in contexts such as tissue repair and mucosal immunity [41,42]. Nevertheless, further studies are required to identify the specific molecular targets of COEB, clarify the biological implications of IL-6 upregulation, and confirm these effects in appropriate in vivo models to fully assess its therapeutic potential.

## 4. Materials and Methods

### 4.1. Reagents

Lipopolysaccharide (LPS, L3012) and Griess reagent (G4410) were purchased from Sigma-Aldrich (St. Louis, MO, USA). MTT (3-(4,5-dimethylthiazol-2-yl)-2,5-diphenyltetrazolium bromide) reagent was obtained from Duchefa Biochemie (Haarlem, The Netherlands). Enzyme-linked immunosorbent assay (ELISA) kits for IL-1β (KET7005), IL-6 (KET7009), and TNF-α (KET7015) were purchased from Abbkine (Wuhan, China). The Prostaglandin E_2_ (PGE_2_) parameter assay kit (KGE004B) was obtained from R&D Systems (Minneapolis, MN, USA). Protein assay dye reagent (5000006) was purchased from Bio-Rad (Hercules, CA, USA). RNAiso Plus reagent was purchased from Takara Bio Inc. (Shiga, Japan). ReverTra Ace™ qPCR RT Master Mix was obtained from Toyobo Co., Ltd. (Osaka, Japan), and BlasTaq™ 2× qPCR MasterMix was purchased from Applied Biological Materials (Richmond, BC, Canada).

### 4.2. Antibodies

Anti-COX-2 (#12282), phospho-NF-κB (#3033), NF-κB (#8242), phospho-IκBα (#2859), phospho-Erk1/2 (#9101), Erk1/2 (#4695), phospho-p38 (#4631), phospho-JNK (#9251), JNK (#9252), phospho-STAT1 (#8826), STAT1 (#9172), phospho-STAT3 (#9145), STAT3 (#30835), phospho-JAK1 (#3331), JAK1 (#3332), phospho-JAK2 (#3776), and JAK2 (#3230) were purchased from Cell Signaling Technology (Danvers, MA, USA). Anti-iNOS (sc-651), IκBα (sc-371), and p38 (sc-535) were purchased from Santa Cruz Biotechnology (Santa Cruz, CA, USA). Anti-α-Tubulin (A01080) was purchased from Abbkine. Horseradish peroxidase (HRP)-tagged secondary mouse and rabbit antibodies (ADI-SAB-300 and ADI-SAB-100, respectively) were purchased from Enzo Life Science (Farmingdale, NY, USA). 

### 4.3. Extraction Process of C. obtusa Bark Extracts

**Figure 6:**
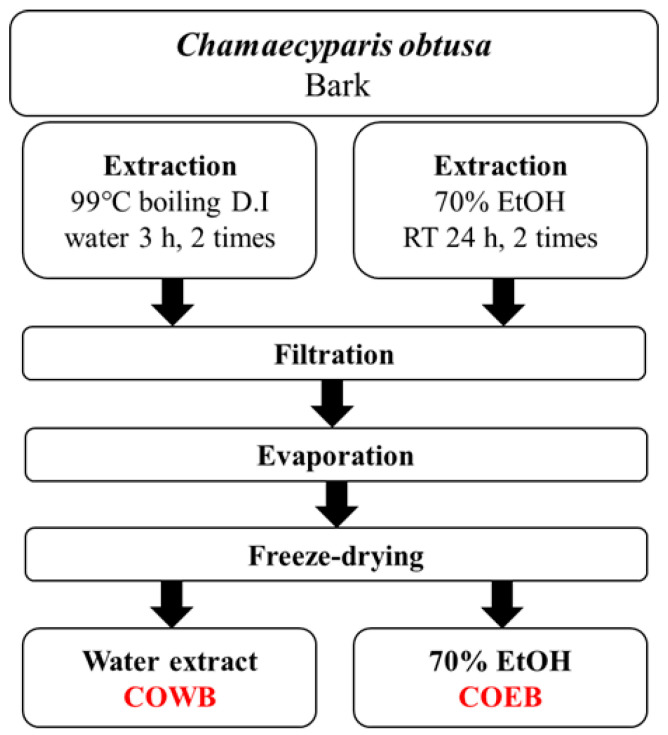
Extraction process of *C. obtusa* bark extracts.

The *C. obtusa* used in this study was obtained from the same source as in our previous research [17,19]. Briefly, the *C. obtusa* samples used in this study were collected from Gwangyang-eup, Gwangyang-si, and Jeollanam-do, Republic of Korea, in March 2020. For the comparative analysis of *C. obtusa* bark extracts based on extraction methods, two types of samples were prepared: a boiling water extract (COWB) obtained by 99 °C boiling *C. obtusa* bark, and a 70% ethanol extract (COEB) obtained by soaking the bark in 70% ethanol. After extraction, each sample was filtered and passed through a filtration system. The filtrates were then concentrated and freeze-dried to obtain the final powdered extracts. The extraction yield of COWB was 3.44%, whereas that of COEB was 5.73%. The overall extraction process is summarized in Figure 6.

### 4.4. High-Performance Liquid Chromatography (HPLC) Analysis

Based on a previously validated method for quercetin analysis using methanol and 0.1% phosphoric acid as the mobile phase [43], high-performance liquid chromatography (HPLC) was performed to identify the active compounds in COWB and COEB. An Agilent Eclipse XDB-C18 column (4.6 × 250 mm, 5 μm) was used at 30 °C. The mobile phase consisted of solvent A (0.1% phosphoric acid in deionized water) and solvent B (methanol). The gradient program is summarized in Supplementary Table S1. The detection wavelength was set at 370 nm using a UV-Vis detector, with a flow rate of 1.0 mL/min and an injection volume of 10 μL. Peak numbers are summarized in Supplementary Tables S2 and S3.

### 4.5. Cell Culture Condition

RAW264.7 (mouse macrophage cell) was obtained from the American Type Culture Collection (ATCC) and cultured in Dulbecco’s Modified Eagle’s Medium (DMEM, Capricorn Scientific GmbH, Ebsdorfergrund, Germany) containing 1% penicillin/streptomycin (Capricorn Scientific GmbH) and 10% fetal bovine serum (FBS, Capricorn Scientific GmbH). These cell lines were incubated in a CO_2_ incubator (5% CO_2_ and 37 °C conditions, PHCbi, Tokyo, Japan).

### 4.6. Cell Viability Analysis

Cell viability was assessed using the MTT assay, based on a previously reported method [44]. Cells were seeded in 96-well plates and incubated overnight in a humidified CO_2_ incubator (PHCbi, Tokyo, Japan). On the following day, the cells were treated with *C. obtusa* bark extracts in a dose-dependent manner and further incubated for 24 h. To assess cell viability, MTT reagent was added and incubated for 2 h. The resulting violet formazan crystals were dissolved in dimethyl sulfoxide (DMSO), and absorbance was measured at 570 nm using a microplate reader (BioTek Instruments, Winooski, VT, USA).

### 4.7. Nitric Oxide (NO) Production

NO production analysis was conducted according to the manufacturer’s instructions (https://www.sigmaaldrich.com 17 July 2025). Briefly, cells were seeded in 6-well plates and incubated overnight in a humidified CO_2_ incubator (PHCbi). On the following day, the cells were pre-treated with *C. obtusa* bark extracts for 2 h, followed by stimulation with LPS for 16 h. The culture supernatant was then collected and reacted with Griess reagent (Sigma-Aldrich) for 5 min in the dark. Absorbance was measured at 570 nm using a microplate reader (BioTek Instruments).

### 4.8. Prostaglandin E_2_ (PGE_2_) Production

PGE_2_ production analysis was conducted according to the manufacturer’s instructions (https://www.rndsystems.com 17 July 2025). Briefly, the culture supernatant was collected using the same procedure as described in the nitric oxide (NO) production assay. PGE_2_ levels were measured using a PGE_2_ parameter assay kit (R&D Systems).

### 4.9. Immunoblotting

Immunoblotting was performed as previously described with minor modifications [45]. Briefly, cells were lysed with 1% Triton X-100 lysis buffer containing protease and phosphatase inhibitors for 10 min on ice, followed by centrifugation at 13,000 rpm for 10 min to remove debris. The resulting supernatant was used for protein quantification using a protein assay dye reagent (Bio-Rad) with bovine serum albumin (BSA) as the standard. Equal amounts of protein were separated by sodium dodecyl sulfate–polyacrylamide gel electrophoresis (SDS-PAGE) and transferred onto nitrocellulose membranes (GE Healthcare Life Sciences, Chicago, IL, USA). Membranes were blocked with 5% skim milk for 1 h and incubated with primary antibodies at 4 °C overnight. On the following day, the membranes were incubated with horseradish peroxidase (HRP)-conjugated secondary antibodies at room temperature for 1 h, followed by detection using an ECL chemiluminescence kit (Biomax, Seoul, Republic of Korea). The signals were visualized on AGFA CP-BU New X-ray film (Agfa-Gevaert N.V., Mortsel, Belgium).

### 4.10. Quantitative Real-Time PCR (qPCR)

Quantitative PCR was performed as previously described with minor modifications [46]. Briefly, total RNA was extracted using RNAiso Plus reagent (Takara Bio Inc., https://takara.co.kr/ 17 July 2025), and cDNA was synthesized using the ReverTra Ace™ qPCR RT Master Mix (Toyobo Co., Ltd., https://www.toyobo-global.com/ 17 July 2025) according to the manufacturer’s instructions, respectively. qPCR was performed using BlasTaq™ 2X qPCR MasterMix (Applied Biological Materials, https://www.abmgood.com/ 17 July 2025), and fluorescence signals were detected with the CFX Connect Real-Time PCR Detection System (Bio-Rad Laboratories). The primer sequences used for mouse *iNOS*, *COX-2*, *IL-1β*, *IL-6*, *TNF-α*, *GAPDH*, and *β-actin* are listed in Supplementary Table S4.

### 4.11. ELISA Analysis of Cytokines

Cytokine production analysis was conducted according to the manufacturer’s instructions (https://www.abbkine.com 17 July 2025). Briefly, cells were seeded in 6-well plates and incubated overnight in a humidified CO_2_ incubator (PHCbi). On the following day, the cells were pre-treated with COEB for 2 h, followed by stimulation with LPS for 8 h. The levels of IL-1β and TNF-α in the collected supernatant were measured by ELISA using commercial kits (Abbkine).

### 4.12. Statistical Analysis

All statistical analyses were performed using Microsoft Excel software (Microsoft Corporation, Redmond, WA, USA). Data are presented as the mean ± standard deviation (SD) from at least three independent experiments. Statistical significance was determined using an unpaired Student’s *t*-test, and *p*-values < 0.05 were considered statistically significant.

## 5. Conclusions

In conclusion, our study highlights the potential of *C. obtusa* bark ethanol extract as a novel natural anti-inflammatory agent. COEB effectively attenuates LPS-induced inflammatory responses in macrophages by inhibiting iNOS and COX-2 expression, independently of classical NF-κB, MAPK, and JAK/STAT signaling pathways. These findings not only provide scientific evidence for the traditional use of *C. obtusa* but also could serve as a novel strategy for its application in the development of functional anti-inflammatory therapeutics.

## Data Availability

No publicly available datasets were generated or analyzed during the current study.

## Supplementary Materials

The following supporting information can be downloaded at: https://www.mdpi.com/article/10.3390/plants14152346/s1, Figure S1: HPLC chromatograms showing the chemical fingerprint profiles of Quercetin; Figure S2: Western blot whole band in Figure 2; Figure S3: Western blot whole band in Figure 4; Table S1: Detailed HPLC condition; Table S2: Peak table of COBW; Table S3: Peak table of COEB; Table S4: The primer sequences of target genes for the qPCR.
