# Supplementary material for: Bark Extracts of Chamaecyparis obtusa (Siebold & Zucc.) Endl. Attenuate LPS-Induced Inflammatory Responses in RAW264.7 Macrophages
Source: Plants (Basel). 2025 Jul 29;14(15):2346. doi: 10.3390/plants14152346 (PMC12349554; doi:10.3390/plants14152346)

Bark Extracts of *Chamaecyparis obtusa* (Siebold & Zucc.) Endl. Attenuate LPS-Induced Inflammatory Responses in RAW264.7 Macrophages

Yong-Jin Kwon *et al.*

Supplementary Table S1. Detailed HPLC condition.

Column	Agilent Eclipse XDB-C18(4.6 x 250mm, 5μm)		
Column temp.	30°C		
Detector	UV-Vis		
Wavelength	370nm		
Flowrate	1.0 mL/min		
Injection vol. and dose	10 μL of 100 mg/ml samples dissolved in 50% MeOH		
Solvent	A: 0.1% Phosphoric acid in DIW<br>B: Methanol		
Gradient condition	Time (min)	A %	B %
	0	85	15
	2	85	15
	20	0	100
	30	0	100
	30.1	85	15
	40	85	15

Supplementary Table S2. Peak table of COBW.

Peak	Ret. Time	Area	Height
1	9.752	1.47805	0.170706
2	12.166	1.30239	0.148284
3	15.787	0.922848	0.0489896
4	16.169	0.877874	0.0965426
5	16.491	0.765026	0.0845471
6	18.387	0.608552	0.0577624
7	19.947	0.801232	0.0821539
8	20.566	1.61758	0.0835629
9	31.136	23.77471	3.05097

Supplementary Table S3. Peak table of COEB.

Peak	Ret. Time	Area	Height
1	9.748	1.43938	0.163617
2	12.17	0.869785	0.123804
3	12.868	1.00312	0.0855512
4	14.872	0.983587	0.105762
5	15.78	0.849643	0.0453734
6	16.174	2.75573	0.305799
7	16.488	1.1616	0.140729
8	18.426	0.839937	0.0647579
9	19.952	0.876099	0.0762713
10	20.566	1.65032	0.146479
11	23.699	0.630141	0.0372597
12	25.716	1.19556	0.136794
13	28.589	4.90623	0.748284
14	30.26	0.699375	0.0897478
15	31.13	24.14903	3.12066
16	31.926	0.815651	0.175246

Supplementary Table S4. The primer sequences of target genes for the qPCR.

Gene name		Sequence (5' -> 3')	Accession No.
COX-2	Forward	TTTGGTCTGGTGCCTGGTC	NM_011198.5
	Reverse	CTGCTGGTTTGGAATAGTTGCTC	
iNOS	Forward	CAGCACAGGAAATGTTTCAGC	NM_001313922.1
	Reverse	TAGCCAGCGTACCGGATGA	
IL-1β	Forward	TTGACGGACCCCAAAAGATG	NM_008361.4
	Reverse	AGAAGGTGCTCATGTCCTCA	
IL-6	Forward	GGTGACAACCACGGCCTTCCC	NM_031168.2
	Reverse	AAGCCTCCGACTTGTGAAGTGGT	
TNF-α	Forward	TATGGCTCAGGGTCCAACTC	NM_001278601.1
	Reverse	CTCCCTTTGCAGAACTCAGG	
GAPDH	Forward	GCAAATTCAACGGCACAG	NM_001411841.1
	Reverse	CACCAGTAGACTCCACGAC	

Supplementary Figure S1. HPLC chromatograms showing the chemical fingerprint profiles of Quercetin.

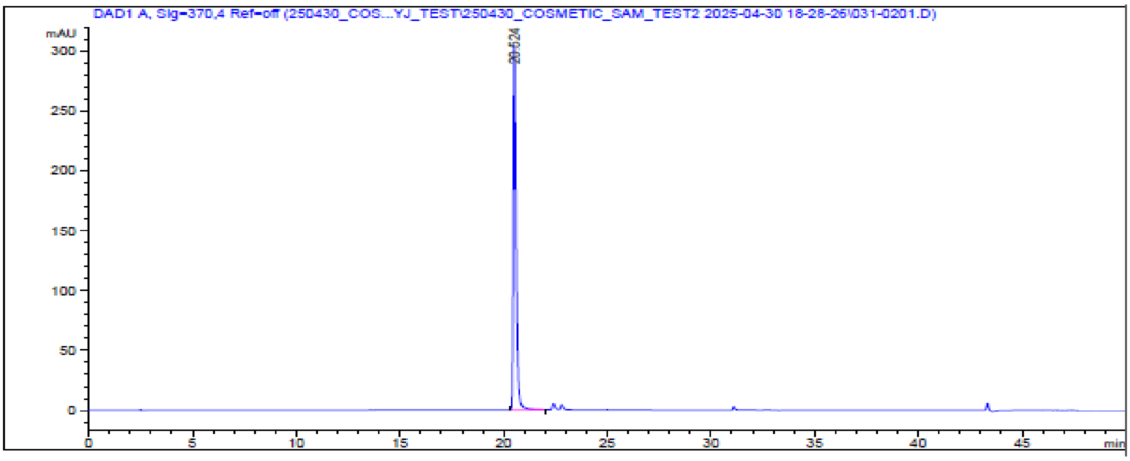

Supplementary Figure 2. Western blot whole band in Figure 2.

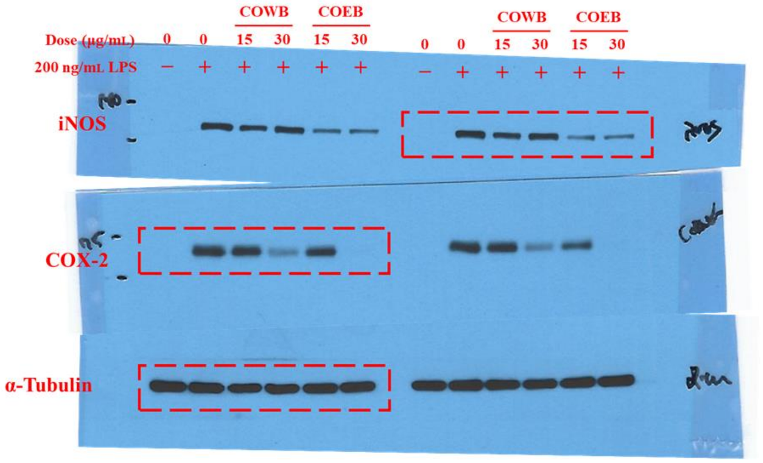

Supplementary Figure 3. Western blot whole band in Figure 4.

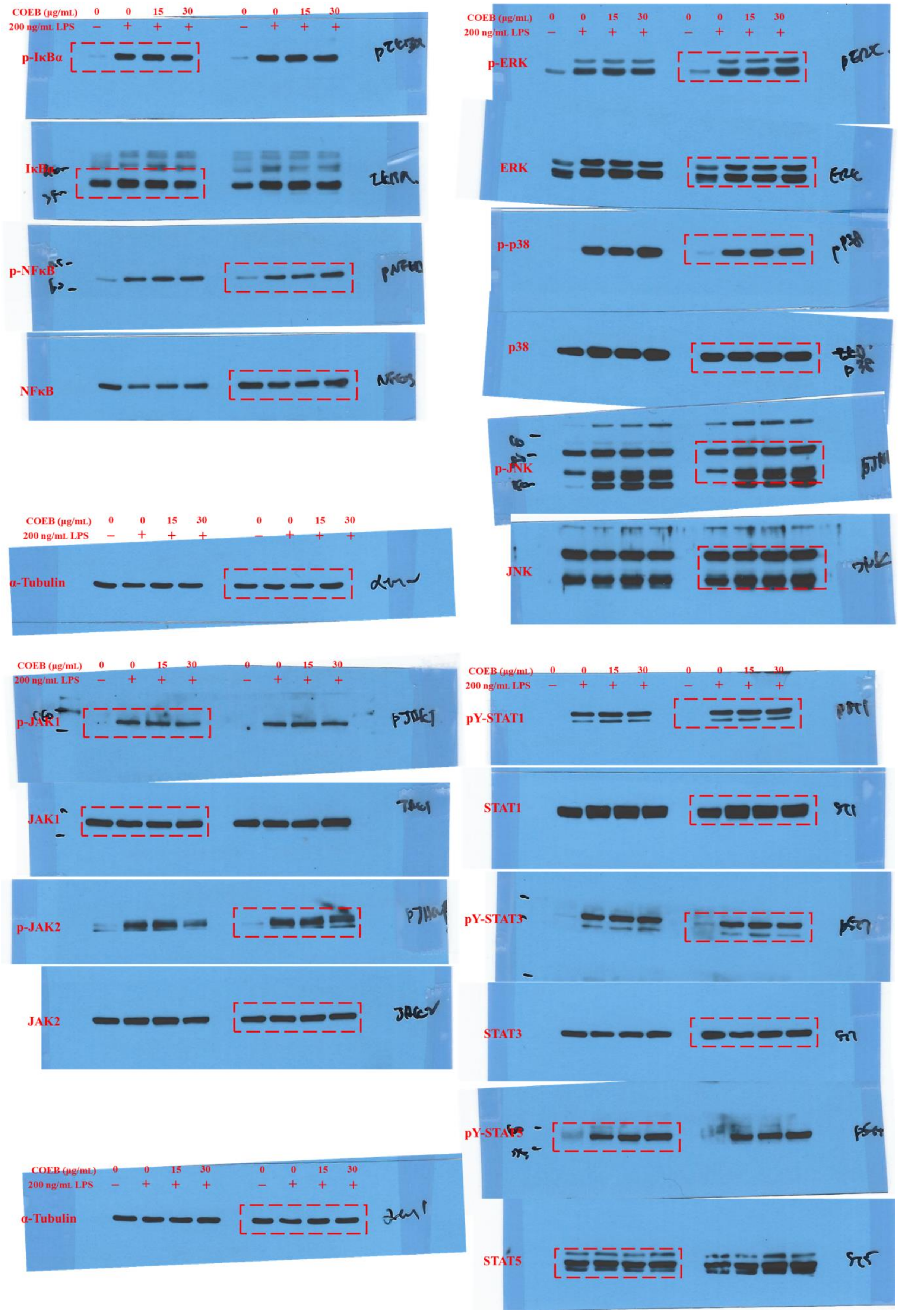

Supplement: Supplementary file 1 [file plants-14-02346-s001.zip › plants-3757253-supplementary.pdf]
